# A Case Report of Sepsis Post Cardiac Catheterization

**DOI:** 10.7759/cureus.32977

**Published:** 2022-12-26

**Authors:** Andrea C Marin, Ankita Prasad, Riddhi R Machchhar, Vraj Patel, Viraj Shah, Kajal Ghodasara, Lee Manchio, Varun Vankeshwaram, Pramil Cheriyath

**Affiliations:** 1 Internal Medicine, Hackensack Meridian Health Ocean Medical Center, Brick, USA; 2 Internal Medicine, Rowan University School of Osteopathic Medicine, Stratford, USA; 3 Internal Medicine, Rajarshee Chhatrapati Shahu Maharaj Government Medical College, Kolhapur, IND; 4 Medicine, Zaporizhzhia State Medical University, Zaporizhia, UKR

**Keywords:** staphylococcus, cardiac catheterization, pci, troponin, nstemi, mrsa, angiography, sepsis

## Abstract

Percutaneous coronary intervention (PCI) and cardiac catheterization are clean procedures done under aseptic precautions, but studies have shown transient bacteremia following the process, mostly involving Staphylococcus. This has many complications, from localized wounds at arterial access sites to endocarditis, mycotic aneurysm, and sepsis, and are associated with high mortality. These may require surgical intervention and prolonged antibiotic use. The risk of acquiring these infections is higher in femoral catheterization than in radial access. This risk also increases in patients with congestive cardiac failure, age 60 and above, and those with diabetes and obesity. Procedural hazards include multiple punctures and leaving the sheath for future access due to the needle tract's colonization. We present a case of sepsis presenting two days after PCI using single puncture radial access and a rapid downhill course.

## Introduction

Diagnostic coronary angiography and percutaneous coronary intervention (PCI) are standard procedures. Even though they are done under strict sterile conditions, asymptomatic bacteremia still occurs in 4-8% of angiographic procedures [1]. Infected angiographic catheters are the most common cause [1]. The infections of post-cardiac catheterization range in severity from localized infections, endarteritis, endocarditis, mycotic aneurysm, and sepsis, as in this patient. Most of these infections are caused by staphylococci, and they may need long-term antibiotic treatment, surgical debridement, or even a new cardiac valve in some cases. Any patient presenting with fevers, chills, or bacteremia within the first four weeks after a procedure should be evaluated for the possibility of infection [2]. In the United States alone, more than 1,000,000 PCI procedures were done in 2013 [3]. Considering the number of procedures, the eventual number of patients who develop sepsis after PCI may be much more than actually documented.

## Case presentation

**Figure 1:**
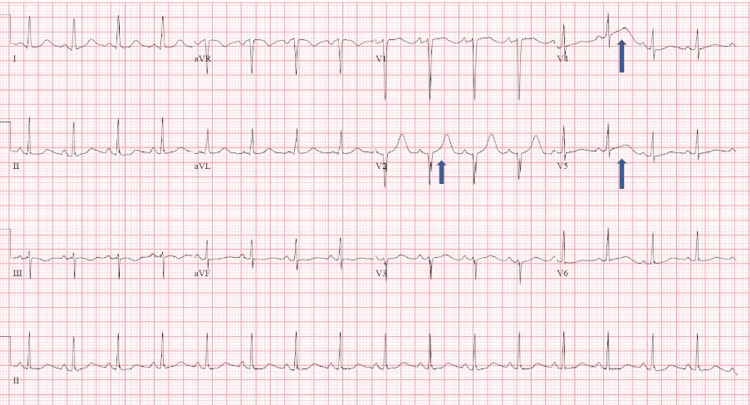
ECG showing ST elevation in V4-V5 (blue arrow) ECG - electrocardiogram

This patient is a 55-year female who presented to the Emergency Department (ED) with nausea and multiple episodes of vomiting for more than 12 hours. She could not drink or eat anything and was exhausted from vomiting. She had no abdominal pain, fever, or bowel and bladder complaints and denied using medications or alcohol. She had no chest pain, shortness of breath, headaches, or changes in vision. Her medical history was significant for hypertension, and cholecystectomy was done 10 years back. She was non-compliant with her antihypertensives. She was taking methadone for 20 years due to a history of opioid addiction. At the presentation, she was conscious, alert, and dehydrated. Her heart rate was 112 beats per minute, her blood pressure was 204/140mm of Hg, her respiration rate was 16/minute, and her oxygen saturation was 96% on room air. The chest, nervous system, and abdomen examinations were within normal limits. An electrocardiogram at admission showed ST elevation in V4 and V5; laboratory investigations showed elevated troponins, 8.15 (<0.04), and she was taken for diagnostic cardiac catheterization (Figure 1).

She was also started on metoprolol, lisinopril, aspirin, atorvastatin, and alprazolam, intravenous ondansetron. These medications helped normalize her blood pressure and control nausea and vomiting. She underwent a cardiac catheterization via radial access, which showed a 70% stenosis in the proximal left anterior descending and apical stenosis of 90%. She had a 90% left circumflex and 90% marginal stenosis, and the right coronary artery was a small nondominant vessel almost completely occluded. The patient had a complex three-vessel disease. Her left ventricular ejection fraction was normal, and a coronary artery bypass graft (CABG) was recommended. She was discharged in a hemodynamically stable state and was advised hospital admission again after two days for CABG evaluation and procedure.

The next day, she returned ned to the ED with back and lower abdominal pain that radiated to the groin on both sides. The pain started a few hours after being discharged from cardiac catheterization and was worse with movement. The pain was more on the left lower extremity. She has no numbness, weakness, tingling, urinary symptoms, nausea, vomiting, chest pain, shortness of breath, or fever. During her ED stay, she started with a fever of 103 Fahrenheit. Her heart rate was 122 beats/minute, respiratory rate was 20 beats/minute, and blood pressure was 78/50 mm of Hg. There was no leukocytosis, but the creatinine went up to 1.9 mg/dl from 1.0 mg/dl two days back. Troponin significantly elevated at 10.25. Lactic acid increased to 6.9, and her urine analysis was normal. Laboratory investigations are in the chart (Table 1).

**Table 1:** Laboratory investigations PTT - partial thromboplastin time, AST - aspartate transaminase, ALT - alkaline transferase

	Admission 1	Admission 2
PTT (25 to 35 sec)	73	32
D-Dimer (<0.5)		41,180
White Blood Count (4.5 to 11.0× 10^9^/L)	4	6.9
Hemoglobin (12.1 to 15.1 g/dL )	10.1	13.8
Hematocrit	33.2	41.8
Platelet Count (1.35-3.17x 10*6/ul)	111	131
Leucocyte count (4.5-11x10*3/ μl)	5400	6000
Neutrophils (40-60%)	64%	53%
Bands, Percent (<10%)	15	33
Glucose (<140mg/dl)	305	382
Blood urea nitrogen (6-24 mg/dL)	31	25
Creatinine (0.6-1.1 mg/dL)	2.76	1.94
Sodium (135-145meq/L)	137	127
Potassium (3.6-5.2mmol/L)	4.6	2.9
Calcium (8.5-10.2 mg/dL)	8	9.1
Alkaline Phosphatase (44-147 U/L)	94	124
Protein Total (6.0-8.3 g/dl)	3.7	6.8
Albumin (3.4 to 5.4 g/dL)	1.5	3
Bilirubin, Total (1.2 mg/dl)	0.9	1.2
AST (10 to 36 U/L)	1573	125
ALT (4 to 36 U/L)	838	50
Lactic Acid (0.5 to 2.2 mmol/L)	3.2	6.9
Troponin (<0.04)	8.19	10.25
Procalcitonin (0.1 ng/mL)	5	65.75

**Figure 2:**
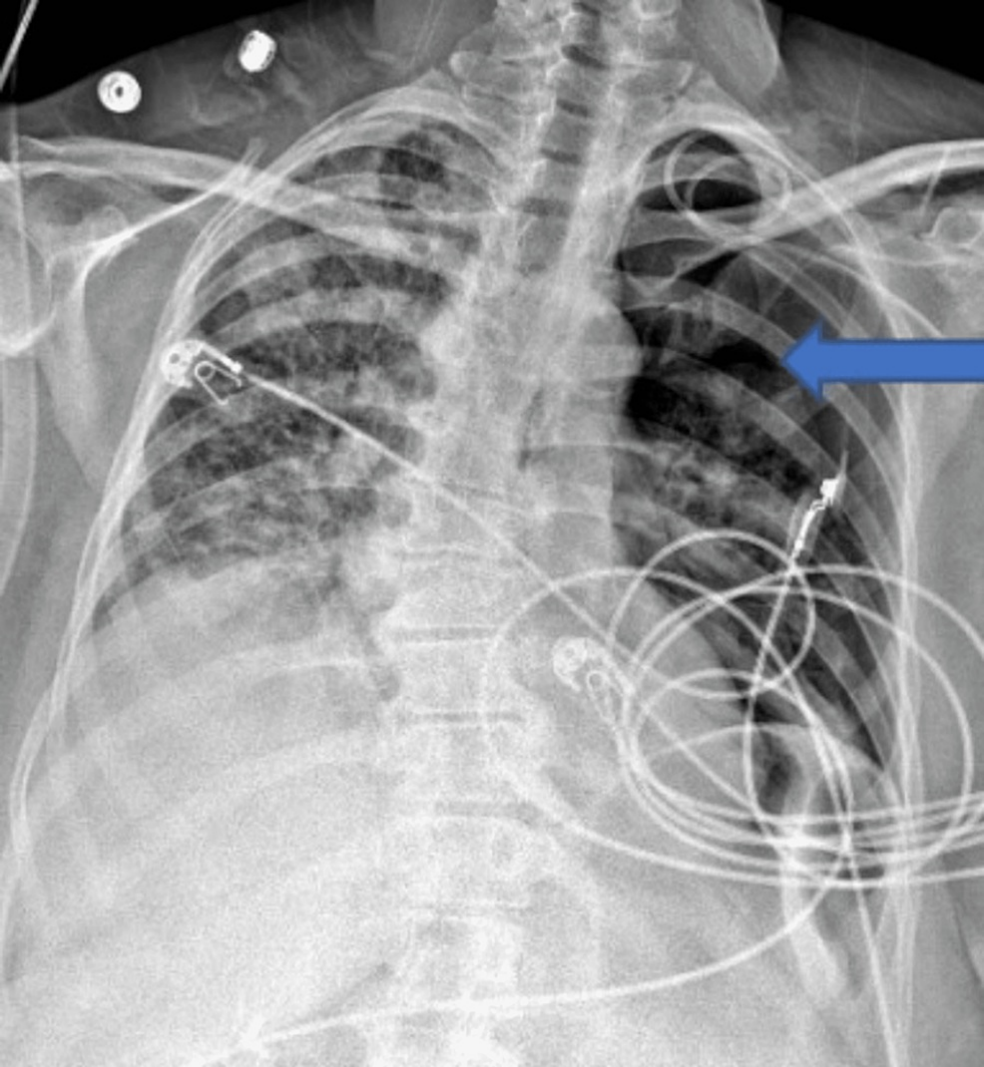
Chest X-ray showing increasing left-sided pneumothorax (blue arrow)

Chest x-ray (CXR) done showed bibasilar atelectasis and left-sided pneumothorax. Sepsis workup, vascular reassessment, and sepsis protocol with antibiotics, piperacillin/tazobactam, and vancomycin started. Her blood cultures later revealed methicillin-resistant *Staphylococcus aureus* (MRSA). Over the day, a rapid response was called as her respiratory status was declining, and she was becoming more tachycardic. A CXR showed worsening left pneumothorax (Figure 2).

She was intubated for airway protection and hypoxemia. She also sustained cardiac arrest but did achieve delayed return of spontaneous circulation (ROSC). A chest tube was inserted in the left pleural space. An emergent triple lumen catheter (TLC) was placed in the right femoral vein. She again became bradycardic and sustained two episodes of cardiac arrest. Unfortunately, the patient did not achieve ROSC after the third code. 

## Discussion

In the United States alone, more than 1,000,000 PCI procedures were done in 2013 [3]. A literature search found that 18% of difficult PCI patients had positive blood cultures immediately, and 12% had positive blood cultures 12 hours later [4]. However, the infections cleared up without any clinical symptoms [4]. Repeated punctures and the sheaths staying in the artery for a longer duration raise the risk of these infections [5]. These risks are also higher in patients with congestive heart failure and age 60 or more, obesity, and diabetes mellitus [6]. Manual compression is often used to stop bleeding after femoral and radial catheterization. They are the most frequently used arterial access sites for PCI because of the ease of achieving hemostasis by manual compression. Twenty cases of septic endarteritis have been documented in PCI with femoral artery catheterization using manual compression to control bleeding [2]. In some studies, anticoagulants like heparin were associated with a higher infection rate [7]. Antibiotic-impregnated catheters have been shown to reduce catheter-associated bloodstream infections significantly [8]. In another study of 21,517 cases of radial artery catheterization, 1.75% of patients had local infections, and 0.45% in 22,355 cases had bloodstream infections [9]. Some studies report that the brachial route is associated with 10 times more infections than the femoral technique (0.6% versus 0.06%) [10].

Sepsis, endocarditis, suppurative pancarditis, stent infection, septic arthritis, epidural abscess, necrotizing fasciitis, and groin wound infection can be complications of infections after coronary catheterization. There is evidence of skin colonization with *S aureus* in the groin of patients with obesity and diabetes, which predisposes them to infections after femoral catheterization [11], and colonization of the needle tract by skin flora increases the risk of sepsis.

Many things perplexed us about this case, including her initial and later presentation and the sudden worsening. The presence of an abscess or pneumonia should have manifested in the workup done at her first admission. However, her laboratory workup and physical examination at the first visit certainly did not point to any of these. There was no other reason to believe such a fatal abscess would develop in the next two days. She had a history of low back and leg pain off and on for some years, in the study by Friedman H, et al. on microbial infections, immunomodulation, and drugs of abuse [12]. It was found that opiate abuse leads to decreased resistance to bacterial and viral infections and altered hypothalamic pituitary adrenal (HPA) axis, corticosteroid production, and cytokine secretion. These abused substances have direct actions on immune cells that seem to be receptor-mediated for all drugs. Thus we explain the presence of lung findings on the rapidly worsening sepsis. These leave us with the invasive procedure being the cause of MRSA sepsis. Our patient had a fulminant course after a diagnostic coronary catheterization with sepsis and death in the next two days. The access was a radial artery, the procedure was without repeated ipsilateral punctures, and no sheath was left. No other factors would predispose the patient to such an outcome. This is a one-of-a-kind case as we have not been able to find any documented case of such fulminant sepsis after a cardiac catheterization, even after an extensive search of the literature using Pubmed, Google Scholar, and Embase.

## Conclusions

Coronary angiography and stenting are widely done and are supposed to be clean procedures but certain procedures and patient-related factors like repeated arterial puncture, leaving arterial sheaths in situ, age over 60 years, diabetes, and obesity predispose to bacteremia. These infections can cause local wounds, endarteritis, endocarditis, and sepsis. These have poor outcomes and usually require prolonged antibiotic therapy and surgical debridement. Staphylococcus skin colonization is the most implicated bacteria and the bacterial introduction may be due to an infected catheter or colonization of the needle tract. The risk of acquiring these infections is higher in the case of femoral catheterization as compared to radial access.
